# Does multidisciplinary rehabilitation of tortured refugees represent ‘value-for-money’? A follow-up of a Danish case-study

**DOI:** 10.1186/s12913-018-3145-3

**Published:** 2018-05-18

**Authors:** Line Bager, Kristian Schultz Hansen, Carit Jacques Andersen, Shr-Jie Wang

**Affiliations:** 1 Danish Institute Against Torture, Bryggervangen 55, 2100, Copenhagen, Denmark; 2 Department of Health Services Research, Institute of Public Health, University of Copenhagen, Oester Farimagsgade 5, 1014 Copenhagen, Denmark; 3 Decisionconsult A/S, Herluf Trolles Vej 243, 5220 Odense, Denmark

**Keywords:** Torture, War, Multidisciplinary intervention, Health economics, Quality of life, Cost-utility, Cost-benefit, Long-term follow-up, Resource allocation

## Abstract

**Background:**

The recent surge of asylum seekers in the European Union (EU) is raising questions about the EU’s ability to integrate newcomers into the economy and into society; particularly those who need specialized services for the treatment of severe trauma. This study investigated whether rehabilitating traumatised refugees represents ‘value-for-money’ (VfM) in terms of intervention cost per health gain and in a long-term and societal perspective.

**Methods:**

The economic evaluation comprised a cost-utility analysis (CUA) and a partial cost-benefit analysis (CBA). The CUA incorporated data on Quality Adjusted Life Years (QALY) for 45 patients who were treated at the Rehabilitation and Research Centre for Torture Victims, Copenhagen, Denmark, in 2001–2004 and followed for up to 2 years, to determine the incremental cost effectiveness ratio (ICER). For the CBA, data was collected for 44 patients who completed treatment between 2001 and 2004 and 44 matched controls on the waiting list, for the patients’ primary health care utilisation, and personal and family labour income from 2001 to 2014. This was analysed to evaluate the Net Social Benefit (NSB) of the programme.

**Results:**

The average cost of treatment was found to be about 32,000 USD per patient (2016 prices) with an average gain in QALY of 0.82. The treatment was cost effective according to the ICER threshold suggested by the National Institute of Health and Care Excellence (UK). At the individual level, the NSB remained negative throughout the study period. However, at the family income level the intervention proved to have been beneficial after 3 years.

**Conclusion:**

The implication of the study is, that providing rehabilitation to severely traumatised refugee families can be an economically viable strategy, considering the economic effects observed at the family level.

## Background

Torture and its consequences have mostly received attention in the academic literature as a socio-political phenomenon with severe psychological ramifications. Nevertheless, torture is also assumed to be financially costly to society, not only in terms of treating the mental and physical sequelae, but also through lost productivity, as torture survivors often struggle to cope with day-to-day work. However, little is known globally or at a country level about the cost of torture to society. The measurement of cost is notoriously difficult, because the parameters involved are not easily defined and the data not easily captured [1]. One study has attempted to model the cost societies incur as refugee-hosting countries; Mpinga and colleagues estimate the economic burden of torture in Switzerland, using estimates of prevalence of torture experience among refugees residing in Switzerland to model the socioeconomic consequences of torture at country level. Their study shows that the effects of torture create substantial economic losses to society. They found that the greater part of this loss is due to the indirect cost (approximately 10 billion CHF) related to the loss of productivity over a period of 30 years. By comparison, direct expenditure related to housing, healthcare, food and education over the same period amounted to roughly 130 million CHF [2]. The authors acknowledge the potentially controversial nature of calculating the cost associated with being a country hosting traumatised refugees, but stress that their findings should be seen within a strong ethical perspective, using the economic argument to support the campaigns for the prevention of torture [2].

The current crises in Syria and elsewhere leave little hope that the number of people traumatised by war and torture will diminish for many years. UNHCR estimates that in 2015 65.3 million people were displaced, the most ever recorded [3]. Many, though not all of them, are forced to flee their own countries and seek asylum in Europe and elsewhere. In 2015, the number of first-time applicants for asylum in Europe increased to almost 1.26 million, more than doubling the number in 2014. This increase was mainly due to applicants from Syria, Afghanistan and Iraq [4]. Among the refugees who come to Europe there will be a need for specialised help to deal with the after-effects of torture and other potentially traumatic experiences. It remains unclear how the current influx of refugees will fare in society, and what impact on the public finances of their host countries their presence will have. This impact will depend on several aspects, such as the age, gender, and skill-levels of the refugees. Research done by the Deutsche Bank and the German Institute for Economic Research (DIW) show that the economic consequences depend on how successful social and economic integration is, and the time-perspective employed in the analyses. Despite large initial cost, these institutions find that investment in refugees is worthwhile in a longer-term perspective [5], with better outcomes modelled for successful integration efforts [6].

The refugee population is far from homogeneous and the specific needs of individuals and their ability to integrate successfully may vary substantially. In particular, refugees who have been exposed to torture and other war-related traumas experience a range of physical and social problems that persist over time [7]. The consequences of torture and war trauma include Post Traumatic Stress Disorder (PTSD), depression, anxiety, and chronic pain, which pose particular challenges for maintaining daily life and functioning [8, 9]. Moreover, trauma has also been shown to affect the family through intergenerational transmission. A person who has suffered torture or war-related trauma may have profound difficulty in maintaining a family role both in relation to his or her spouse and in terms of parental responsiveness and role function [10–12]. Not only do intimate partners of survivors display an elevated level of psychiatric symptoms and feelings of loneliness, among other things [11], but studies show that parental PTSD and depression is strongly correlated with child distress [13, 14]. Little is known about the relation between short-term health outcomes for refugees and the longer-term socioeconomic outcomes for the individual as well as the family. So far, the evidence we do have suggests that victimisation of individuals place an extra financial burden on the individual and the family. Family members might employ various coping strategies to address an increased burden of care, including taking up debt, stop going to school or work to care for the victim in the family [15, 16].

Research evaluating rehabilitation programmes has tended for many years to have an exclusively clinical focus, especially in the specific area of rehabilitation for torture and war survivors [17]. One aspect of ensuring access to good quality rehabilitative care for traumatised refugees also involves providing evidence of the societal cost of torture, and on the cost-effectiveness and the long-term economic impact of providing rehabilitative services [18]. However, despite their importance, the economic implications of torture have not been a research priority [19] and only few have attempted to document the economic viability of providing specialised care for tortured and war-affected populations [20]. In Denmark, care for tortured refugees is available at specialised clinics across the country. However, no systematic effort is currently in place to screen refugees for torture trauma at the point of arrival to the country [21]. Therefore the specific issues and challenges torture survivors face, may either not be addressed at all, may be managed in the Danish health care system at large or, if referred to a specialised clinic for refugees be addressed in this context. Moreover, despite public demand for documenting the effect of the resources spend on rehabilitative efforts and the long-term socioeconomic outcome for this group, no systematic effort has been carried out at this stage.

The present study addresses the gap in knowledge about the economic effects of rehabilitation programmes by evaluating a specific multidisciplinary rehabilitation programme for torture survivors, from the point of view of its economic viability. The rehabilitation programme was provided for a severely traumatized group of refugees living in Denmark. It is unique in that it addresses the socio-economic consequences of providing multidisciplinary rehabilitation by combining data on short-term self-reported health improvements with longer-term economic data covering labour income and expenditure on health services. The study is to our knowledge the only of its kind combining both a cost-utility analysis (CUA) and a partial cost-benefit analysis (CBA), using actual rather than modelled data, to answer the question of whether rehabilitation for survivors of torture and war represent ‘value-for-money’ (VfM) in a societal perspective. This information will aide policy makers in the allocation of expenditure in the Danish health system as well as provide crucial feedback to the specialised clinics who have direct contact with, and knowledge of, this population’s concerns and needs.

## Methods

### Two-pronged approach to estimating ‘value-for money’ (VfM)

**Fig. 1:**
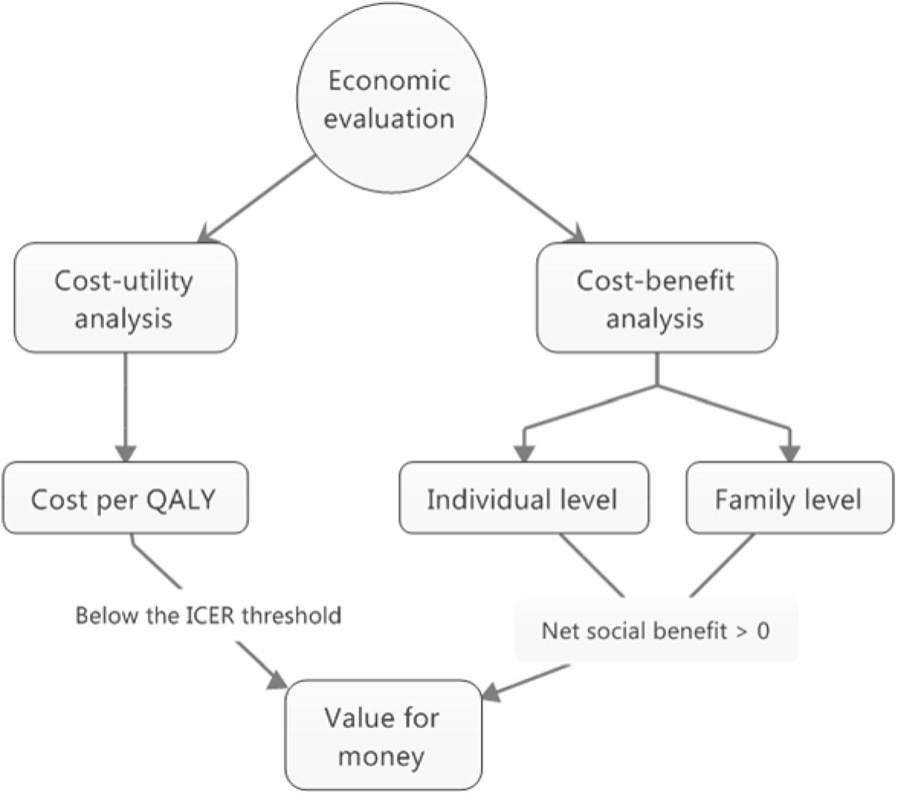
Conceptual model, two-pronged approach to VfM

Evaluations of health interventions face the risk of excluding important impacts when focusing exclusively on individual clinical outcomes. Consequently, the true effect of the programme is underestimated as this occurs [22]. To take the complex comorbidity torture survivors present into account, and the multifaceted nature of the rehabilitation programme, the study employed a two-pronged approach by combining a cost-utility and a partial cost-benefit analysis, when estimating VfM (See Fig. 1 below). This approach included a short-term perspective, using data from self-reported quality of life (over 23 months) and a longer-term societal perspective, based on population register data on labour income and health care consumption in the primary sector over a 14-year period (2001–2014). To provide further nuances and qualify our analysis, we included labour income not only for the individuals in the study population but subsequently also for their families. Figure 1 below depicts the analytical approach in the study, illustrating the decision criteria by which an intervention can be categorised as providing VfM. Each analysis estimates VfM as compared to a pre-determined decision criterion. In this study, the result of the cost-utility analysis is compared to a threshold value, while the cost-benefit analysis expresses the intervention’s net contribution to society as a monetary value.

### Datasets

The calculations are based on records of a course of treatment provided for refugees in the Rehabilitation and Research Centre for Torture Victims (now DIGNITY – Danish Institute Against Torture), in Copenhagen, Denmark. The effect of the treatment is captured using various clinical measures, monitoring the process from visitation through the follow-ups [23]. The treatment provided was individualised and multidisciplinary in nature, consisting of sessions with psychologists, doctors with different specialisations (neurologist, psychiatrist and rheumatologist), physiotherapists, nurses, and social workers, and was delivered as either individual or group therapy (for women). The composition of the treatment, i.e. the number and types of sessions with professionals, varied per the individual patient’s needs as determined by the treatment team and the patient. The cost of treatment and other services provided was found using DIGNITYs financial records from 2001, when the study was initiated. The treatment composition at patient level was recorded and used to calculate an average treatment cost. The average treatment cost was used in both analyses and is the same for both the CUA and the CBA. However, the CUA and the CBA had different time perspectives and used different outcomes measures. The necessary datasets were obtained from various sources as illustrated in Table 1.

**Table 1:** Sources of data

	Cost-utility analysis		Cost-benefit analysis	
	Cost	Utility	Cost	Benefit
Variable/outcome	Cost of treatment	Quality Adjusted Life Years (QALY)	Cost of treatment Primary health care consumption	Employment Individual labour income Family labour income
Source of information	Financialaccounts (organisation)	Questionnaire data: WHOQOL-Bref [27]	Financial accounts (organisation) Population registers (Statistics Denmark)	Population registers (Statistics Denmark)
Time period	2001 (representative year for individual treatment cost)	2001–2004 Patients were enrolled in the study at differing times and data collected at baseline, 9 and 23 months.	2001–2014	2001–2014

#### Dataset: Cost-utility analysis

The key source of data for the CUA consisted of the cost of treatment and monitoring data on improvements in health-related quality of life. The calculation of the initial cost of treatment will be described in detail below. Mental health and health-related quality of life were assessed before the multidisciplinary rehabilitation was carried out, and again at 9 and 23 months in a previous study by Carlsson and colleagues [23]. From this previous study, which consisted of a comprehensive evaluation of mental health and health-related quality of life, we used the data from one outcome measure on self-reported quality of life as recorded for a group of 45 individuals (the original sampled population was 69) who had no missing data at baseline, 9 and 23 months’ follow-up. The original study was designed as a pre-post study, and changes in quality of life was measured against the baseline.

#### Dataset: Cost-benefit analysis

Data from Danish population-based registers over the period 2001–2014 and the cost of treatment formed the basis for this calculation. The Danish registers, both administrative and research-focused in nature, contain substantial information on all individuals residing permanently in Denmark. Residents of Denmark can be identified through a unique personal identification number which can be linked to the different registers, making the registers highly relevant for longitudinal studies of socioeconomic and welfare outcomes [24] From *Statistics Denmark*, which is a key supplier of register data, we obtained data on labour income and health care consumption in the primary sector for 44 treated and 44 untreated individuals as well as labour income for their families.

### Study populations

**Fig. 2:**
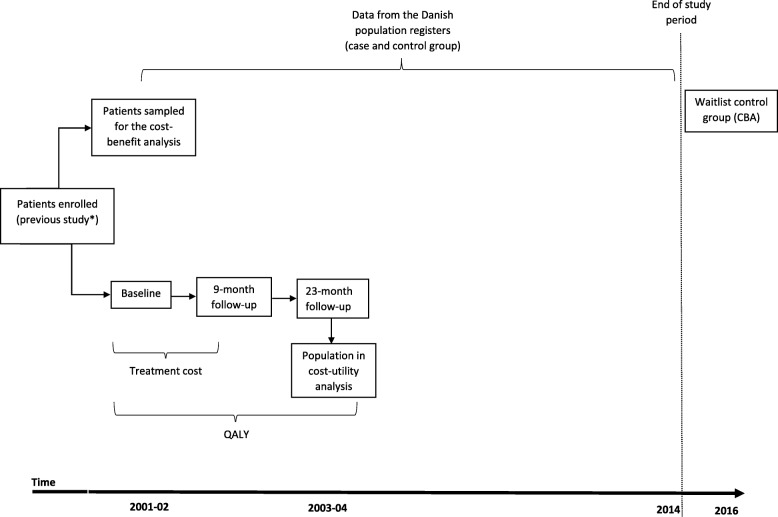
Study overview

All individuals included in this study were patients treated at the Rehabilitation Centre and Research for Torture Victims in Copenhagen and who all had a history of torture and war trauma. The individuals were referred to the clinic by their general practitioner. See Fig. 2 for the study overview.

#### Cost-utility analysis

From the original study of 69 individuals, only 45 had complete data at 23-months follow-up and the calculations on cost of treatment and quality of life is therefore based on these 45 individuals. These 45 individuals were treated at in the period from 2001 to 2004. This sample consisted predominantly of individuals with Iraqi origin (66.7%) and the group was majority male (66.7%). At the 23 months’ follow-up, this group had been in treatment for an average of 14.3 months (SD = 6.98) and received 60.6 treatment sessions on average (SD = 43.19) [23]. From the original study, only data on quality of life and information on treatment composition for the individual patients were used (individual versus group-based, and number and types of sessions received).

#### Cost-benefit analysis

The study population included in the CBA was also a sample from the original case-study with 69 individuals [25]. The potential pool of individuals to be included in the CBA was larger than for the CUA, as the analysis was not dependent on a complete set of monitoring data. This was because the data could be obtained from the population registers for all patients in the original case study through the patients’ civil registration number.

**Fig. 3:**
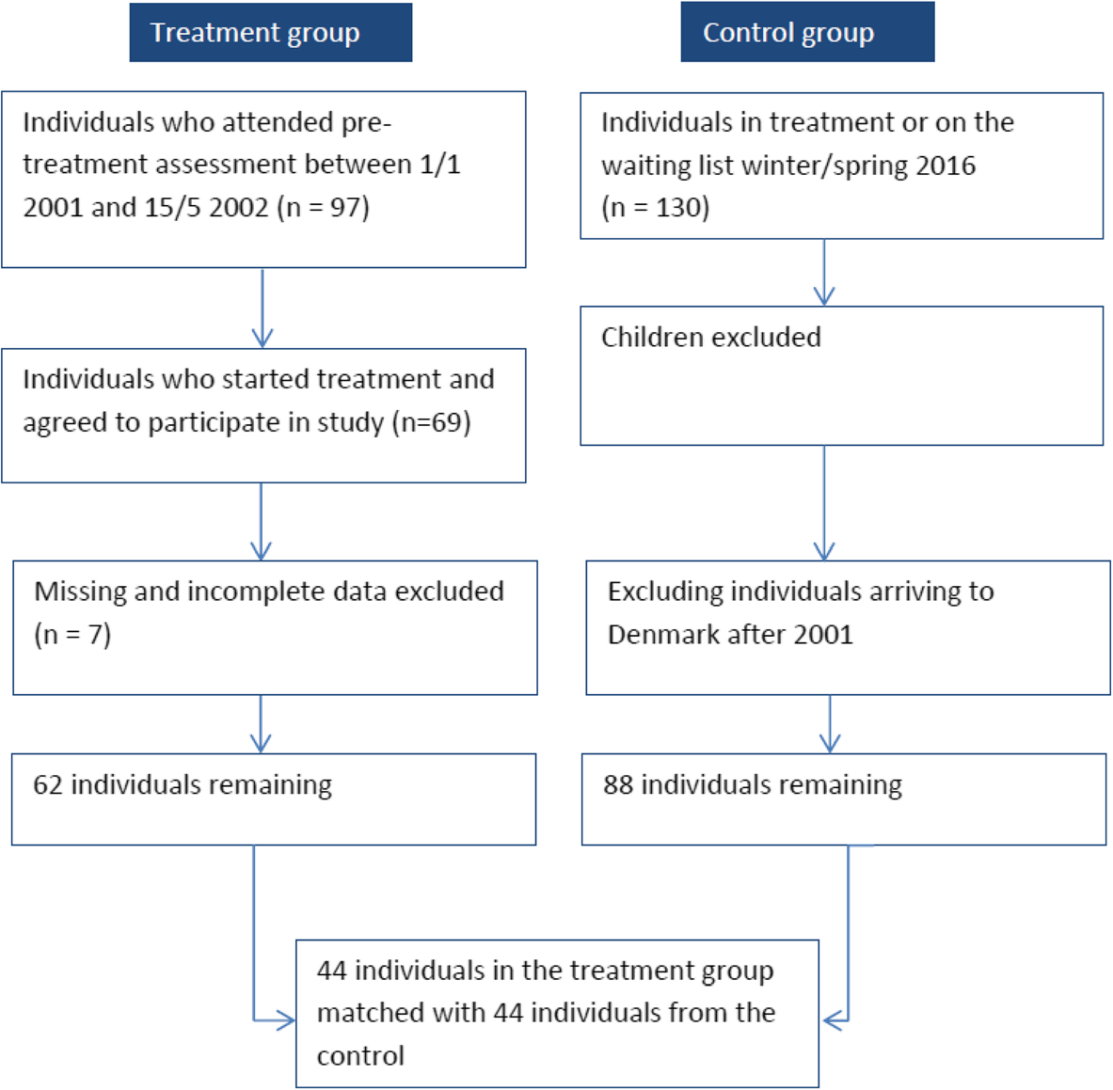
Sampling procedure (CBA)

For the CBA, a control group was needed. Finding a suitable control group for a study over a long period was challenging as it would have been highly unethical to select identified torture survivors and not offer them immediate treatment. A comparison group was therefore assembled with patients who were in treatment or on the waiting list at the time of the sampling procedure (winter/spring 2016). The sampling procedure for the CBA is illustrated below in Fig. 3. While 69 individuals were included in the original case study, only 62 had sufficient data allowing for the matching procedure. Similarly, out of the 130 individuals on the waiting list or in treatment at the time of the sampling procedure, only 88 were eligible to be included. The 62 cases were matched with the 88 controls using propensity score matching, without replacement. A total of 44 treated individuals were matched with 44 control individuals on age, gender, country of origin, time of arrival to Denmark and on torture and/or war trauma. Matching individuals on the latter variable occurs indirectly, as it is what qualifies this group of individuals to receive treatment at DIGNITY, as a clinic granted special status in this area by the Ministry of Health in Denmark.

For the expansion of the CBA, family members of the study population were included. The family as a unit is defined by *Statistics Denmark* and includes individuals living in the household with the same family ID number. This includes children who are defined as children (biological or adopted) of at least one of the adults, 24 years old or younger, not married or divorced and living at home.

### Data analysis

#### Cost of treatment

i. Step-down costing
  DIGNITYs internal financial accounts provided the information for calculating treatment cost. Standard recommendations for step-down costing were followed [26]. The allocation of facility and overhead cost were allocated to departments using a spatial allocation criterion (square metres used) as well as the number of staff in the department, depending on the cost category. The cost per session type (medical, nursing, psychological, group-based, social work, physiotherapy) was then calculated using the total salary for each professional group and the total number of services produced for each type of treatment.
ii. Micro costing
  After the cost of different types of sessions had been determined, this information was combined with information on the actual treatment received by the members of the study group. This was possible since for the patients in the study group, the number of each type of treatment session had been recorded. This enabled the calculation of the treatment cost to be completed at individual level. The information was used to calculate the average treatment cost which forms the basis for the cost calculations of both the CUA and the CBA. The treatment record available however, did not distinguish psychological and medical assistance from each other so these were grouped. The same was the case for physiotherapy, nursing, and social support sessions. In calculating the cost of sessions, the former groups were allocated a higher cost than the latter. The average treatment cost was consequently constructed based on the number of high and low-fee sessions received by the study group.

### Quality adjusted life years (QALY)

For the CUA, the outcome measure was Quality Adjusted Life Years (QALYs). QALYs were calculated using results for the WHO Quality of Life questionnaire, brief version (WHOQOL-Bref) [27] and the approach developed by Hwang and Wang [28]. The WHOQOL-Bref is a quality of life assessment instrument which includes 24 facets covering four domains and in addition, two facets related to general health. The four domains relate to physical health (pain, sleep, daily activities), psychological health (self-image, negative thoughts, positive attitudes, self-esteem, mentality, memory concentration), social health (relationships, social support, sex life) and environmental health (access to financial resources, safety, health services and social services, the physical living environment, opportunities to acquire new skills and knowledge, recreation). The 26 questions are rated on a five-point Likert scale. The WHOQOL-Bref is a disease-independent, validated instrument for population studies [29]. It was suitable for this particular group of patients as it is culturally sensitive [27] and has been validated in a Danish context, including for psychiatric patients [30].

For each facet, the question scores for the domain were summed up after reversing the score for the negatively phrased questions (Q3, Q4 and Q26). The mean score was then found and multiplied for each domain. This process transformed the WHOQOL-Bref score into a score directly comparable to that of the longer instrument, WHOQOL100. The total score for quality of life could then be created by summing the domain scores. For the purpose of calculating QALYs, we assume that the utility assigned to death is zero and that the survival time is unaffected by the treatment, which is an approach used for chronic, non-fatal diseases [31]. It means that Quality Adjusted Life *Months* (QALMs) can be found though a trapezoidal approximation where,

$$ QALM=0.5\ast \left(Q\left({t}_0\right)+Q\left({t}_1\right)\right)\ast {n}_1+0.5\left(Q\left({t}_1\right)\right)\ast {n}_2 $$

The quality of life data was collected at n_1_ and n_2_, which in this study corresponds to 9 and 23 months’ follow-up. To convert QALMs into QALYs, the result was divided by 12.

### Incremental cost effectiveness ratio (ICER)

The incremental gain in utility in this case is expressed as compared to the baseline and used to find the Incremental Cost Effectiveness Ratio (ICER) of the treatment. This ratio is then compared to an ICER threshold, typically defined in policy by a national public health body. As Denmark and the other Scandinavian countries do not operate with an explicit or implicit ICER threshold [32], this study refers to the range proposed by the National Institute of Health and Care Excellence (NICE) in the UK, which suggests a most plausible ICER threshold range of £20,000–30,000 [33], below which an intervention can likely be considered cost-effective. Mathematically the ICER is defined as:

$$ ICER=\frac{C_1-{C}_0}{E_1-{E}_0} $$

Where C_1_ represents the cost of the intervention and C_0_ is the cost of the comparator. E_1_ is the effect measured for the intervention and E_0_ correspondingly the effect of the comparator. In the present study, C_1_ is the treatment cost and E_1_ is the QALY as measured at follow-up. C_0_ is zero as no comparator treatment was available. Similarly, E_0_ is the baseline quality of life measured, which is assumed to represent what would have happened without treatment. As no comparison group is available from the original study this calculation therefore expresses the assumption that quality of life would have remained constant over the period and that no additional cost or savings are incurred in this no-treatment alternative scenario.

### Net social benefit (NSB)

The Net Social Benefit (NSB) is a measure quantifying the net contribution to society of an intervention or programme. To evaluate the net contribution of the rehabilitation programme, the concept of the NSB is used as the decision criterion on whether the intervention is a good investment. The NSB is found by subtracting social cost (C) from social benefits (B) [34]. All cost and benefits are measured as a monetary value;

$$ NSB=B-C $$

The benefits include income gained through employment (labour income), excluding any transfer payments. Transfer payments refer to a transfer of surplus between individuals or groups in society and is thus a matter of distribution of existing resources [35]. That is, social benefits provided by the Danish welfare system to the patients in the study are not included in the analysis as such transfer does not constitute an actual resource consumption [26]. The cost includes the cost of treatment and the cost of consumption of health care services in the primary sector. To establish the net cost and benefit associated with the treatment, the values for the control group were subtracted from the treatment group. This was done for each year and subsequently cumulatively, over the 14-years to find the NSB for the study period. The entire cost of treating 44 individuals was then ascribed to 2001 although in reality the treatment of those individuals would have taken place over a few years. In addition, to capture effects at the family level, the NSB was recalculated replacing individual labour income with data on family labour income. To make sure any differences in family labour income is not solely due to differences in family size, the age distribution in the two groups was investigated.

## Results

### Cost analysis

The cost allocation has been illustrated below in Table 2, showing fixed and variable cost. Treatment specific salary, that is, salary of the therapist and those directly involved in the treatment of patients, amounts to just over 5 million DKK. Non-salary fixed and variable cost accounted for the remaining 4.5 million DKK. The ratio of salary to overhead was 0.88. For every 1 kr. spent on treatment in terms of direct salary expenses, another 0.88 kr. was added to account for the general overhead.

**Table 2:** Cost allocation, 2001

Cost categories (DKK)		
Fixed cost (DKK)		
Capital cost	Rent and inventory	615.267,00
Variable cost (DKK)		
Overhead	Utilities, building services	403.373,00
	Office supplies	233.506,00
	Administration	547.491,00
	Reporting and board activities	349.256,00
	Organisational development	449.044,00
Support	Reception	235.800,00
	Library and canteen	291.666,00
	IT	300.928,00
Department specific	External assistance (dentist)	163.800,00
	Supervision and transport	188.967,00
	Salaries (support staff)	692.974,00
	Salary (all treatment staff)	5.080.659,00
Total (DKK)		9.552.731,00

Not all patients who received treatment at the clinic in 2001–02 were included in the study. Therefore the actual cost of the intervention does not equal that of the above table. The number and type sessions the patients in the study received were recorded and the total salary expense of the treatment group calculated as shown in Table 3. The total intervention cost was found by multiplying the direct salary related expenses with the ratio established above of 0.88. The average cost of treating an individual amounted to 166,112.6 kr. per patient (2001 prices), which corresponds to the total intervention cost divided by the number of patients in the study.

**Table 3:** Intervention cost

	**Session type**			
	Medical or psychological assistance	Physiotherapy, nursing, social support, counselling,	Women’s group	Visitation
Number of sessions	1250	1178	43	45
Cost per session (DKK)	2099	984	1796	2538
Cost of sessions, total (DKK)	2,624,871	1,159,751	77,229	114,246
Total salary cost of intervention (DKK)	3,976,098			
Total intervention cost incl. Overheads (DKK)	7,475,065			

### Cost-utility analysis

Table 4 summarises findings of the changes in QALY from baseline to 9 and 23 months’ follow-up, as found using the WHOQOL-Bref questionnaire. The instrument demonstrated good internal consistency for both baseline, 9 and 23 months’ follow-up with Crohnbach’s alpha values of 0.931, 0.933 and 0.915 respectively. The average gain in QALY per client was found to be 0.82, with considerable variations observed in the four different domains. The largest increase in QALY is seen in the environmental domain with a gain of 1.16 while a loss of 0.74 QALY per patient was observed in the social domain.

**Table 4:** Results, QALY (*N* = 45)

Follow-up times		Physical	Mental	Social	Environmental	Total
Baseline (control)	0–8	344,63	330,81	397,56	366,83	1.439,83
	9–23 months	462,80	442,13	542,48	512,65	1.960,05
	total	807,43	772,94	940,04	879,48	3.399,88
Group	0–8	346,21	329,64	391,27	385,14	1.452,26
	9–23 months	468,71	453,40	515,49	546,76	1.984,38
	total	814,92	783,04	906,76	931,90	3.436,64
∆QALY	0–8	1,57	- 1,17	- 6,29	18,30	12,43
	9–23 months	5,92	11,27	- 26,99	34,12	24,33
	total	7,49	10,10	- 33,28	52,42	36,76
∆QALY per patient	0–8	0,03	- 0,03	- 0,14	0,41	0,28
	9–23 months	0,13	0,25	- 0,60	0,76	0,54
	total	0,17	0,22	- 0,74	1,16	0,82

### Incremental cost effectiveness ratio

In the absence of a comparison treatment, the average treatment cost was divided by the average gain in QALY to establish the ICER;

$$ ICER=\frac{C_i-0}{\left({QALY s}_{23}-{QALY}_9\right)+\left({QALY}_9-{QALY s}_0\right)}=\frac{166,112.56 kr}{0.82\; QALY}=202,576.3 kr\; per\; QALY\kern0.17em gained $$

Adjusting this to January 2016 prices, using the consumer price index (CPI) [36] the ICER is:

$$ ICER=\frac{99.4}{76.7}\ast 202,576.3 kr=262,530.4 kr\; per\; QALY\kern0.17em gained $$

Where 99.4 is the CPI for January 2016 and 76.7 is the CPI for January 2001.

The National Institute for Health and Care Excellence (NICE) in the UK has for over a decade referred explicitly to a range in which the ICER threshold could lie. This threshold is between £20,000–£30,000 or roughly 190,000 kr. – 290,000 kr.^1^ It is noted that NICE has not adjusted this range since it first published the guidelines in the early 2000s. The result as shown above, regardless of whether the treatment cost is displayed in 2001 or 2016 prices, indicate that the treatment is within, although in the upper region of what would be considered cost-effective, using the NICE guidelines [33]. In several other countries, public bodies or institutions have proposed ICER thresholds; In the Netherlands, the council for Public Health and Health Care has proposed a maximum ICER threshold of €80,000, corresponding to roughly 595,000 kr. per QALY gained [37].

### Cost-benefit analysis

#### Balance of covariates

The CBA involved two groups, the treatment and control group, which were matched on 4 covariates (age, gender, country of origin, time of arrival to Denmark). The distributional balance of these were checked using *chi*-square test for the categorical values and paired *t*-test for the continuous values. As is illustrated by the summary table (Table 5), all four covariates display non-significant difference in the distribution of these between the treatment and control group. Moreover, there was no significant difference between the size and age distribution in the families of the two groups.

**Table 5:** Matching - balance of covariates

Match type	Count	Covariates	Pearson *chi*-square (*p* value)	Paired *t*-test (*p* value)
Exact	1	Gender	0.338	
Fuzzy	43	Country of origin	0.464	
Unmatched	18	Arrival to Denmark	0.233	
		Age		0.456

#### Net social benefit (NSB)

**Fig. 4:**
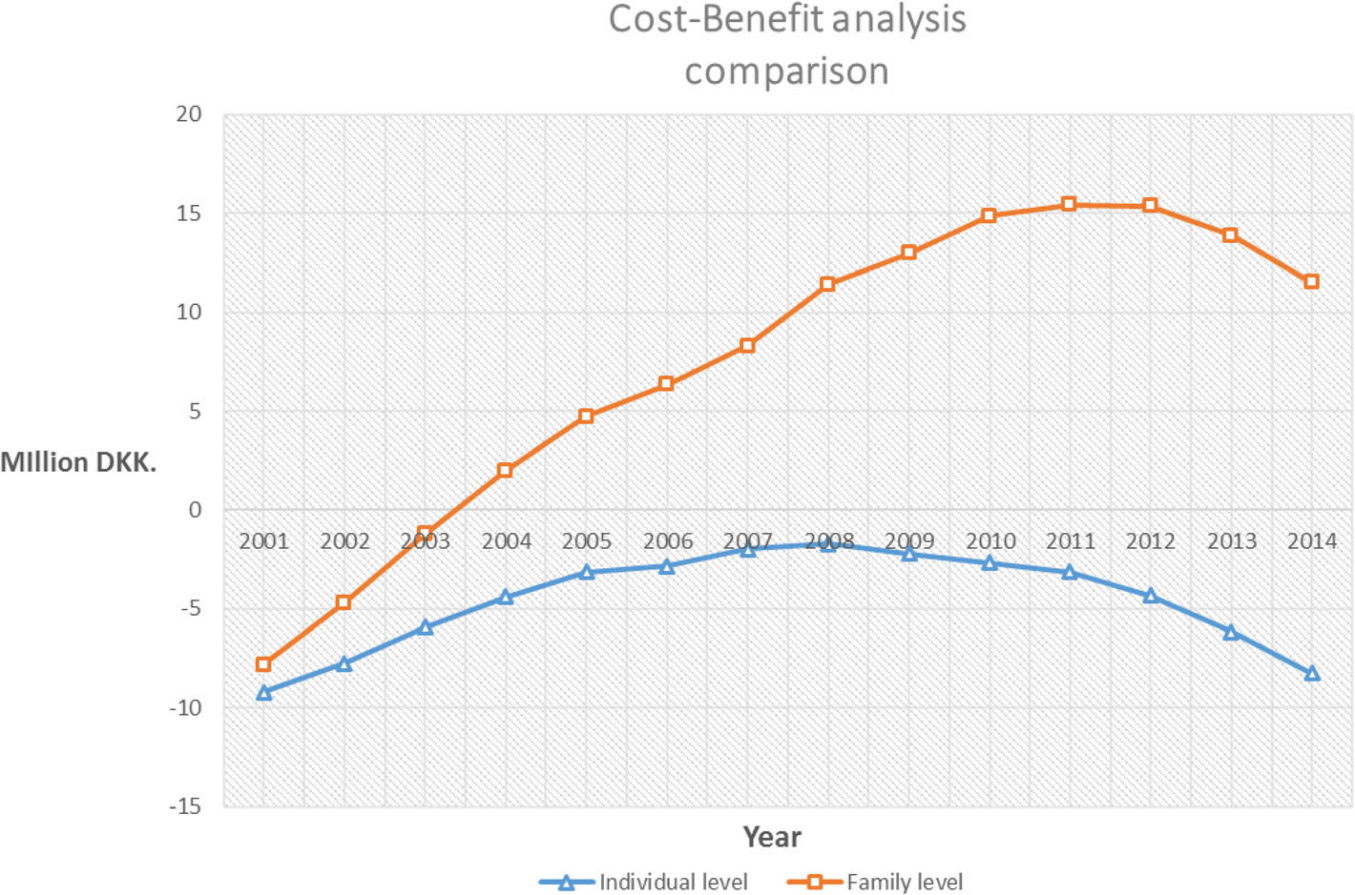
Comparison of CBA for individual and family level

Table 6 and Fig. 4 show the result from the analysis from the individual client perspective as well as the family perspective. The cost for both groups includes the costs associated with utilisation of primary health care services. For the patients from the original study, the treatment group, the full treatment cost in January 2016 prices have been allocated the year 2001. The final column in the individual and family category respectively in Table 6, displays the NSB over the period (cumulative).

**Table 6:** Summary of CBA (DKK 2016 prices)

	Individual				Family			
Year	Net cost	Net benefit	NSB	NSB	Net cost	Net benefit	NSB	NSB
	C_case_-C_control_	B_case_-B_control_	Per year	Cumulative	C_case_-C_control_	B_case_-B_control_	Per year	Cumulative
2001	−9,608,486	422,944	−9,185,542	-9,185,542	-9,608,486	1,817,001	−7,791,485	−7,791,485
2002	27,578	1,427,666	1,455,245	−7,730,297	27,578	3,061,108	3,088,687	−4,702,797
2003	2034	1,827,058	1,829,092	−5,901,204	2034	3,485,928	3,487,963	−1,214,834
2004	9675	1,489,537	1,499,212	−4,401,991	9675	3,210,567	3,220,243	2,005,408
2005	− 1615	1,287,029	1,285,414	−3,116,576	−1615	2,700,899	2,699,284	4,704,693
2006	−39,980	330,460	290,479	−2,826,097	−39,980	1,677,093	1,637,112	6,341,805
2007	−60,595	901,123	840,528	−1,985,569	− 60,595	2,016,465	1,955,869	8,297,675
2008	−103,962	392,095	288,132	−1,697,436	−103,962	3,225,204	3,121,241	11,418,916
2009	−116,958	−403,512	− 520,471	−2,217,907	− 116,958	1,648,385	1,531,426	12,950,343
2010	−91,569	− 353,164	− 444,733	−2,662,641	− 91,569	1,984,194	1,892,625	14,842,968
2011	−114,644	− 351,721	− 466,366	−3,129,007	− 114,644	684,060	569,415	15,412,383
2012	−116,577	−1,080,575	− 1,197,152	−4,326,160	− 116,577	44,460	−72,117	15,340,265
2013	−67,216	−1,755,809	−1,823,026	−6,149,187	− 67,216	− 1,420,965	−1,488,182	13,852,083
2014	− 127,280	−1,958,948	−2,086,228	−8,235,415	− 127,280	− 2,210,873	− 2,338,153	11,513,930
Total	−10,409,599	2,174,184	−8,235,415		−10,409,599	21,923,530	11,513,930

Figure 4 compares the cumulative NSB for the two different levels in the analysis. The results show a marked difference between the CBA taking the individual as a starting point versus that for the family as whole. For the individual level analysis, the curve that illustrates the cumulative NSB has an upward trend towards break-even. This is, however, never reached (the line never intersects the x-axis) and the NSB remains negative throughout the study period. A quite different picture is observed when family rather than individual labour income is plotted. From 2004 onwards, the NSB is positive and although it displays the same reverse in trend, it is far from being as pronounced as at the individual level. The reason for this change in trend will be discussed later.

## Discussion and conclusion

The rehabilitation of survivors of torture is of great importance, not only for the individuals concerned but for the societies in which they are living. DIGNITY in Denmark is a clinic offering specialised multidisciplinary rehabilitation for torture survivors. In the process of evaluating and testing such programmes, not only information on efficacy but also effectiveness is needed. This ‘value-for-money’ (VfM) perspective is increasingly important in a health care setting with limited resources, where decisions must be made on what health interventions to provide. This paper describes the procedure of two analyses to assist such decisions on VfM.

Torture is a complex phenomenon, which affects social, economic, physical and mental dimensions of the everyday lives of survivors and their families. In this study, we employed a societal perspective, investigating the cost-effectiveness and long-term economic benefits of providing multidisciplinary rehabilitation. The CUA was calculated based on a group of 45 individuals who had been in the rehabilitation programme in the period 2001–2004. The measured gain in QALYs showed that the intervention was cost-effective within the upper limit of the NICE ICER threshold, which is an internationally recognised comparator of health care cost. As it is our interest to elucidate changes in the relationship between the survivor and society, we disaggregated the gain in QALYs to each of the four domains (physical, mental, social and environmental) measured by the WHOQOL-Bref questionnaire and a more differentiated picture was revealed (see Table 2). The outcome data for the CUA covers roughly 23 months and the results for the psychological and physical domain shows little change. Considering the level of chronicity of the health problems survivors of torture and war experience, it is likely that data collected over a relatively short period might not reveal functional changes. The environmental domain on the other hand, showed a much larger increase in QALYs than the other domains (1.16 as compared to 0.82 overall). This domain is of special interest to this study as it provides information about the individuals functioning in society. It is well-known that PTSD and other symptoms are not static but can present themselves in recurrent relapses. Aggravation and escalation of symptoms can occur as environmental stressors interact with the trauma history [38–40]. The environmental domain encompasses 8 questions covering topics ranging from how safe the respondent feels in daily life (Q8), the financial situation (Q12), the possibility of taking up leisure activities (Q14) and access to health care services (Q25), among others [27]. The calculation of the ICER for the environmental domain is shown separately below. As is demonstrated, the result is below the suggested NICE threshold (190,000–290,000 kr.) of what is to be considered cost-effective, specifically for the integration into a new environment.

$$ {ICER}_{environmental}=\frac{166,112.56 kr}{1.16\; QALY}=143,200.5 kr\; per\;{QALY}_{environmental} $$

This is also the case in 2016 prices (185,500 kr. per QALY_environmental_). While the environmental domain displayed a positive gain in QALYs, the result was quite different for the social domain. Disaggregated, the social domain accounted for a 0.74 loss in quality of life, on the surface indicating that the participants fare worse after treatment. However, this effect may be partly due to the qualities of the instrument. The WHOQOL-Bref questionnaire has the most question in the environmental domain (8) and the fewest in the social domain (3). Therefore, the changes in the score for the social domain makes this domain more sensitive to varying responses. Furthermore, the social domain covers the respondent’s satisfaction with his or her personal life (Q20), sex life (Q21) and support from friends (Q22) [27]. While these questions represent important aspects of well-being, the specific focus of this study was how the treatment could potentially improve the study population’s functioning in society, which is why we placed emphasis on the environmental domain.

We found very few studies in the literature that could contextualise the results of the CUA and no study specific to our population in a high-income setting; one study in Australia found that trauma-focused cognitive behavioural therapy (TF-CBT) in combination with sertraline was superior to TF-CBT alone, non-directive counselling and the non-treatment alternative for sexually-abused girls with PTSD or PTSD and depression. The ICER the authors found amounted to AU$22,263 (approx. 102,400 kr.) which is well below the Australian ICER threshold of AU$50,000 (approx. 230,600 kr.) [41]. Similarly, in a study of US war veterans with PTSD, prolonged exposure theory proved more efficient than sertraline along with an ICER of [42].

For the CBA in the second stage of the study, we included objective, long-term data that could reveal the socio-economic outcomes for the study population over a longer period. An interesting picture was revealed, showing that the treatment never breaks-even in the individual level analysis while a substantial positive NSB is seen over the 14-year study period, when taking the family as the unit of analysis (see Fig. 4). Looking at the values for the NSB at both the individual level and at the family level, a trend is observed where the cumulative NSB over the period first increases and then decreases. Several aspects need to be highlighted in this respect; the individuals in the treatment group performed better with respect to individual income from 2001 to 2008. However, after this point the income started to decrease again. A similar trend is observed for the controls, though the decline from 2008 onwards is less marked. The trend is repeated for family income where the families of the treated individuals performed better than the families of the controls except for the years 2013 and 2014. The data on the expenses related to primary health utilisation show a mixed picture in which from 2006 onwards the expenses of individuals in the treatment group display an increasing trend while those of the controls remain relatively constant. As the study population is small, it is difficult to say whether the increase in primary health care expenditure is due to a change in health seeking behaviour induced by the rehabilitation or if the treatment and control group were systematically different in health seeking behaviour from the beginning.

Both individual and family income demonstrate that 2008 is a pivot point in terms of the overall trend (Fig. 4). The results should be seen in the context of the wider societal context and 2008 represent the onset of the financial crisis that also impacted the Danish economy. A study by *Statistics Denmark* for a larger sample of migrants and refugees from some of the same countries (Iraq, Afghanistan and Turkey) confirms the trend towards a peak in 2008 [43]. In *Statistics Denmark’s* analysis, the declining trend in employment of refugees from Iran and Afghanistan and economic migrants from Turkey is also partly ascribed to the economic crisis. In such a situation, the individuals in our study population, who are vulnerable in various ways, might be particularly exposed to a contraction of the economy; at the onset of the financial crisis the study population is on average 43 or 44 years old, they suffer from health issues, generally have a low-skill level and thus it is likely that they faced challenges in retaining their job or finding jobs once the economic situation in Denmark improved after the recession.

### Sensitivity analysis

In the calculation of the ICER, the cost per gained QALY amounted to 262,530 kr. in current prices. Of this cost, 122,864 kr. was overhead related cost and the remaining 139,666 kr. being the direct salary related cost. There are two considerations in this composition of this overall cost per QALY gained; One consideration is the accuracy in the estimation of the overhead share and the other being the difference in treatment composition, causing a variation in the salary component of the treatment cost. There was a degree of uncertainty about the overhead expenses as the evaluation was carried out some years after the treatment programme, and information about the organisational structure, inventory and staff was no longer complete. Moreover, as the treatment composition varied considerably, it is also worth considering whether the population included in the original study can be assumed to have received a course of treatment comparable to that of other patients in the clinic. To test the decision uncertainty, both inputs to the overall treatment cost were varied by 20% in either direction. This variation did not impact the conclusion in relation to the reference ICER threshold obtained from NICE. That is, the change did not move the established ICER below the NICE ICER threshold lower boundary of £20,000, or above the upper boundary of £30,000.

We also tested how the NSB would change if we equalised the two groups’ health expenditure in the primary sector as well as the outcome for a scenario where the financial crisis was assumed not to have happened. Despite the treatment and control group showing different trends, the actual magnitude of the primary health care expenditure did not significantly alter the outcome of the analysis either at individual level (NSB remained negative) or at the family level (NSB remained positive). However, under the assumption that the financial crisis in 2008 was the sole responsibility of the change in the income of the two groups, we kept the income level for this year constant from 2008 to 2014, which was exactly enough to reach break-even in 2014. Under this scenario, the NSB at the individual level became positive.

### Does rehabilitation for traumatised refugees represent ‘value-for-money’?

The overarching goal of this analysis was to determine whether the specific rehabilitation provided at RCT (now DIGNITY) in 2001–2004 represents ‘value-for-money’. This question was answered through a CUA, looking at cost per QALY and a partial CBA that calculated the NSB, based on individual and family income and primary health care expenditure. The shorter-term focus of the CUA illustrated the cost-effectiveness of the multidisciplinary intervention at the individual level. The cost-effectiveness of the intervention might be greater if the ability of the intervention to support the study populations’ coping with everyday life and integration into society is emphasised. This is indicated by the disaggregated results for the environmental domain.

The partial CBA also indicated a positive effect; at the family level, the productivity gains by family members led to a positive NSB after only a couple of years and this gain persisted over the course of the study period. Therefore, based on the included parameters, the multidisciplinary intervention provided at DIGNITY from 2001 to 2004 was shown provide ‘value-for-money’ and to be an economically sustainable strategy. The results also show that the chosen study design can highlight important dynamics otherwise not revealed; in this study, it was done by taking a broader perspective, including multi-level variables and transgenerational effects when evaluating multidisciplinary interventions for torture or war survivors. By considering these parameters, the study has contributed an alternative perspective to focusing on symptoms. Many of the symptoms suffered by torture survivors are chronic in nature [44–46], so their persistence may not adequately represent the change of functionality in society that the multidisciplinary rehabilitation provides.

### Strengths, limitations and further research

There are several restrictions in the study that limit the strength of the conclusions. The effectiveness study carried out in 2001–2004, providing quality of life data to the CUA, was designed as a pre- and post-treatment study. Therefore, there is no control group and instead the baseline was used as the comparator. It is uncertain to what extent this baseline estimate represents an accurate depiction of what would have happened in the absence of treatment. However, and referring again to the long-term and persistent physical and mental health consequences of war and torture, it might be reasonable to presume that no significant improvement would have happened in the absence of treatment over the 23-month period and so the baseline data for quality of life is a good approximation of the alternative.

Disaggregating the QALY into the different domains helped us to gain more insight into the results of the rehabilitation programme. However, whether it is theoretically sound to do so is debateable. Another point that has been criticised is the reliance on an ICER threshold, as it might not adequately represent society’s willingness to pay for the programme. It has to be emphasised that the NICE threshold is a politically defined threshold, grounded in economic theory but not with an unproblematic transfer to practice [37]. For this study, however, the ICER threshold as suggested by NICE, was necessary as no other cost-effectiveness analysis of an appropriate comparator treatment was available and as the ICER threshold has not been defined in the Danish context.

The CBA investigated socioeconomic outcomes for the study group over a 14-year period (2001–14), using data on individuals’ primary health care use, their labour income and the families’ labour income obtained from the Danish population registers. Other impact categories could have been of relevance but was not possible to include in this study and therefore should the CBA be regarded as a partial CBA. As this group suffer from serious mental health issues, memory bias is an important concern when including self-reported data. Hence, the use of long-term and objective register data is a key strength of the study as it supports and further qualifies the results measured at the clinical level. While the long-term perspective in the partial CBA provides valuable insight into economic outcomes that cannot usually be captured in shorter-term clinical follow-ups, this also presents challenges to finding a suitable control group which introduces a potential bias. To minimise this, it was decided that enrolling the patients currently in treatment or on the waiting list, represented the best approximation with respect to the potential covariates. Creating a sample from the general refugee population would not have allowed us to match participants on torture or war-related trauma exposure. The members of both the treatment and control groups were all eligible for DIGNITY rehabilitation, and had arrived in Denmark at the same time. Nevertheless, the members of the control group had accessed DIGNITY’s services much later. The reason behind this difference is not known, but it may be a difference between the two groups that could result in bias. Members of the control group could have had a different health seeking behaviour, or there may have been differences in the referral system.

When evaluating the effect of health interventions we often risk underestimating the true effect as a too narrow focus is employed [22]. The welfarist approach underlies the CBA and several aspects have been criticised; Among others, it is based on a theoretical compensation principle, the implicit inclusion of income in the decision-making process (with social willingness to pay) and the central notion that health is valued in monetary terms (see [47] for a discussion on these issues). The concept of QALY is part of an effort to move towards non-welfarist or extra-welfarist approaches and is inspired by Amartya Sen’s Capability approach focusing on freedoms of ‘beings and doings’ [22, 47]. However, those who advocate for a more holistic approach to evaluations of health and social interventions argue that although health is maximised in this perspective, QALY as a concept has limited capacity to capture non-health and functional changes especially for chronic patients [48]. This is a highly valid point also in the light of the complex intervention evaluated here as well as the chronic nature of the symptoms the patients in this study experience. At this point, nonetheless, no adequately tested instrument is available or was not available at the time the clinical data was recorded.

Yet, in this study we have attempted to address the challenges by including a range of variables and approached the ‘value-for-money’ perspective from two angles, using both self-reported, short-term and clinical data in combination with longer-term objective data on socioeconomic outcomes. Separately, the CUA and partial CBA show that the intervention is a sound economic strategy. Yet, the strength of the study is the combination of methods, data-sources and time perspective, which underline the conclusion: Rehabilitating severely traumatised refugees can generate economic benefits, not only to the individuals but also their families and society. Future research should take steps to include a larger sample size and more variables for a full CBA, such as data on hospital care and expenses related to social services or crime. Furthermore, as time passes the possibility to obtain more detailed socioeconomic information from the population registers on children of traumatised refugees becomes possible, allowing for an expansion of the analyses of intergenerational effects of trauma.

## Abbreviations

CBA: Cost-benefit analysis
CHF: Swiss franc
CPR: Personal identification number
CUA: Cost-utility analysis
DIGNITY: Danish Institute Against Torture
DIW: German Institute for Economic Research
DKK: Danish Kroner
EU: European Union
ICER: Incremental Cost-effectiveness Ration
NICE: National Institute Against Torture
NSB: Net Social Benefit
PTSD: Post-traumatic stress disorder
QALM: Quality-adjusted life month
QALY: Quality-adjusted life year
SD: Standard deviation
UK: United Kingdom of Great Britain and Northern Ireland
UNHCR: United Nations High Commissioner for Refugees
VfM: Value-for-Money
WHOQOL-Bref: World Health Organization Quality of Life questionnaire, brief version

## Footnotes

1 The range refers to the currency exchange before the United Kingdom’s European Union membership referendum on 23rd June 2016.
