# Correction to: does multidisciplinary rehabilitation of tortured refugees represent ‘value-for-money’? A follow-up of a Danish case-study

**DOI:** 10.1186/s12913-018-3276-6

**Published:** 2018-06-13

**Authors:** Line Bager, Kristian Schultz Hansen, Carit Jacques Andersen, Shr-Jie Wang

**Affiliations:** 1 Danish Institute Against Torture, Bryggervangen 55, 2100 Copenhagen, Denmark; 2 Department of Health Services Research, Institute of Public Health, University of Copenhagen, Oester Farimagsgade 5, 1014 Copenhagen, Denmark; 3 Decisionconsult A/S, Herluf Trolles Vej 243, 5220 Odense, Denmark

## Correction

Following publication of the original article [1], the authors reported a correction in the name of one of the authors.

The name in the original article is:

Shr-Jie Sharlenna Wang.

The correct name is:

Shr-Jie Wang.

## References

[1] Bager L (2018). Does multidisciplinary rehabilitation of tortured refugees represent ‘value-for-money’? A follow-up of a Danish case-study. BMC Health Serv Res..

